# Bortezomib-induced peripheral neuropathy: from molecular mechanisms to clinical management

**DOI:** 10.3389/fphar.2025.1719384

**Published:** 2025-11-26

**Authors:** Yuting Yan, Yanqi Song, Quan Zhang, Lingwei Hu, Aidi Wang, Honglei Guo, Zhen Wang, Lin Ma, Baoshan Liu

**Affiliations:** 1 Tianjin University of Traditional Chinese Medicine, Tianjin, China; 2 Tianjin Medical University General Hospital, Tianjin, China; 3 Tianjin Medical University, Tianjin, China

**Keywords:** bortezomib-induced peripheral neuropathy, neurotoxicity, neuropathic pain, clinical syndromes, pathogenesis, therapeutic approaches

## Abstract

Bortezomib (BTZ) is a first-generation proteasome inhibitor that has shown significant efficacy in the treatment of multiple myeloma (MM), providing significant therapeutic benefits without severely compromising overall patient health. However, one of the most common and debilitating side effects associated with BTZ therapy is bortezomib-induced peripheral neuropathy (BIPN). This condition is the leading complication observed in patients undergoing BTZ treatment and has a profound impact on treatment regimens, often necessitating dose reductions or alterations in the dosing schedule. Despite the widespread recognition of BIPN, therapeutic options remain limited. Consequently, further exploration of the molecular mechanisms underlying BIPN is crucial to identify potential therapeutic targets. Establishing standardized, effective treatment strategies is also essential to improve patient outcomes. This review seeks to provide an in-depth overview of the current research on BIPN, covering its clinical presentation, potential pathophysiological mechanisms, and emerging therapeutic strategies. The aim is to offer valuable insights to support the development of novel therapeutic approaches and enhance clinical management of this challenging side effect.

## 1 Introduction

Bortezomib (BTZ) is a synthetic dipeptide salt of borate and a reversible inhibitor of the 20S proteasome complex (Kane et al., 2003; Richardson et al., 2003). The introduction of BTZ has significantly altered the course and prognosis of multiple myeloma (MM), making it possible for MM patients to achieve complete remission or very good partial remission. Nevertheless, bortezomib-induced peripheral neuropathy (BIPN), which is the most frequent and severe side effect of BTZ therapy, has become an increasing concern in the management of MM (Argyriou et al., 2014; Argyriou et al., 2008; Park et al., 2013; Salat, 2020; Yamamoto and Egashira, 2021; Yang X. X. et al., 2024). Clinical studies reported that BIPN occurs in 15%–57.2% of MM patients, with severe neuropathy (grade 3–4) observed in 1%–30%, depending on the study, assessment methods, and patient populations (Dimopoulos et al., 2017; Durie et al., 2017; Moreau et al., 2016; Palumbo et al., 2016; Peng et al., 2015; Sonneveld et al., 2012; Tacchetti et al., 2014; Velasco et al., 2019; Zinzani et al., 2012). Although several theories with empirical support tackle the point, the causes of BIPN are multifactorial and not fully understood, it remains a potentially persistent adverse event that can lead to long-term objective neurological dysfunction (Li et al., 2020), thereby reducing the quality of life, weakening physical function, causing delays or dose reductions in chemotherapy, and in some cases even interrupting treatment, imposing a huge economic burden (Selvy et al., 2021). Owing to the paucity of high-quality, consistent evidence, therapeutic options for preventing BIPN or treating established neuropathy remain limited. Accordingly, in depth investigation of BIPN pathophysiology and the development of mechanism-based therapies are urgently needed to improve prevention and symptom management.

## 2 Clinical syndromes: clinical features, diagnosis and risk factors of BIPN

### 2.1 Clinical features

Patients typically present with abnormal sensations in the extremities, with initial symptoms often manifesting in the feet. Key symptoms include pain, numbness, stiffness, and muscle spasms (Velasco et al., 2019; Selvy et al., 2021; Geisler, 2021; Mattar et al., 2024). Many patients experience moderate to severe pain, often described as stabbing or burning, which can persist for years after treatment cessation, significantly impairing their quality of life (Ibrahim and Ehrlich, 2020; Michalova et al., 2023; Tsai et al., 2021; Yan et al., 2021). Severe BIPN can result in muscle atrophy, diminished ankle reflexes, and reduced proprioception, leading to foot drop, ataxia, and gait disturbances, all of which can impair daily functioning (Yamamoto and Egashira, 2021; Michalova et al., 2023; Singh et al., 2020; Vaxman et al., 2022). BTZ may also induce autonomic neuropathy, manifesting as orthostatic hypotension and alterations in sexual and urinary function, though such cases are relatively rare (Burgess et al., 2021; Zhang, 2021). The median recovery time for BIPN is approximately 3 months, which is relatively favorable compared to PN induced by other chemotherapeutic agents. Improvement in PN to a lower NCI grade was slower in newly diagnosed MM patients, with a median recovery time of 1.9 months, whereas previously treated patients showed a median recovery time of 3.6 months (Velasco et al., 2019; Dimopoulos et al., 2011; Yang Y. et al., 2024). Additionally, some patients may experience paradoxical worsening or develop new chemotherapy-induced peripheral neuropathy (CIPN) after BTZ discontinuation, a phenomenon known as “coasting”, which can lower survival rates, particularly in children and adolescents (Tay et al., 2022; Zajaczkowska et al., 2019).

### 2.2 Clinical diagnosis

To date, there is a lack of established diagnostic standards for BIPN, which leads to a frequent underestimation due to patient underreporting and insufficient assessment by physicians (Addington and Freimer, 2016). The clinical instruments employed to diagnose and evaluate the severity of neuropathy can generally be classified into three key categories: patient-reported outcome measures, comprehensive scoring systems that incorporate functional assessments, and evaluations focused on quality of life (Burgess et al., 2021; Park et al., 2019). The optimal approach combines patient-reported symptom severity with objective clinical assessment scales and neurophysiological testing (Starobova and Vetter, 2017).

The National Cancer Institute Common Terminology Criteria for Adverse Events is currently the most widely used assessment tool in clinical practice. However, this assessment is primarily clinician-driven, relatively subjective, and lacks objective data, which may lead to significant variability depending on the evaluator (Park et al., 2019; Cavaletti et al., 2010). The Eastern Cooperative Oncology Group criteria and the World Health Organization Neurotoxicity Scale are also widely utilized. Comprehensive instruments such as the Total Neuropathy Score (TNS) integrate patient-reported symptoms, physical examination findings, vibratory perception thresholds, and nerve conduction studies. The TNS captures the severity of BIPN more comprehensively (Burgess et al., 2021; Alberti et al., 2021). Functional assessments typically involve patient-reported questionnaires that measure the impact of neurotoxicity on quality of life and identify specific activity limitations experienced by patients. These assessment tools are frequently customized for particular cancer types, such as the Functional Assessment of Cancer Therapy/Gynecologic Oncology Group Neurotoxicity scale, to evaluate neurotoxicity-specific impacts. The European Organization for Research and Treatment of Cancer Quality of Life Questionnaire for Chemotherapy-Induced Peripheral Neuropathy and the European Organization for Research and Treatment of Cancer Quality of Life Questionnaire, *etc* (Park et al., 2019; Alberti et al., 2021). Additionally, median level of pain intensity and the total score on the McGill Pain Questionnaire can be used to assess moderate to severe BIPN-related neuropathic pain (Bechakra et al., 2018).

Additionally, neurophysiological assessments, including nerve conduction studies, quantitative sensory testing, skin biopsy, corneal confocal microscopy, and SUDOSCAN, are valuable tools for detecting alterations in BIPN symptoms and assessing the effectiveness of treatments (Allegra et al., 2022; Argyriou et al., 2019; Bechakra et al., 2020; Cata et al., 2007; Cocito et al., 2015; Kandula et al., 2017; Lauria and Lombardi, 2007; Thawani et al., 2015). Despite this, diagnostic criteria for BIPN remain undefined, highlighting the urgent need for additional research aimed at establishing a standardized and comprehensive assessment framework.

### 2.3 Risk factors

The onset of BIPN is usually associated with factors such as the drug dosage, dosing frequency, and route of administration (Argyriou et al., 2008; Li et al., 2020). During bortezomib therapy, peripheral neuropathy occurred in 21% of patients receiving 1.0 mg/m^2^ per dose and in 37% receiving 1.3 mg/m^2^. With continued treatment, the incidence increased gradually through the fifth cycle (cumulative exposure >30 mg/m^2^), peaked at approximately 42–45 mg/m^2^, and then approached a plateau in cumulative risk (Dimopoulos et al., 2011; Richardson et al., 2009a). A *post hoc* analysis of a phase III trial found that administering the drug twice a week was linked to a significantly higher rate of severe BIPN compared to once-weekly dosing (28% vs. 8%). In both groups, with similar cumulative BTZ doses, the regimen administered at a lower intensity resulted in a shorter recovery time (2.3 vs. 3.2 months) (Bringhen et al., 2010). Moreover, a meta-analysis revealed that once-weekly BTZ administration decreased the occurrence of PN of any grade, including grade ≥3, when compared to biweekly BTZ treatment. Nonetheless, additional clinical research is required to confirm the efficacy and role of the weekly BTZ regimen (Hu et al., 2017). A randomized trial revealed that subcutaneous injection resulted in a 15% decrease in the incidence of BIPN across all grades and a 10% reduction in grade 3 or 4 BIPN compared to intravenous administration (Moreau et al., 2011). Another meta-analysis of 16 studies involving 2,575 patients indicated a lower risk of BIPN and comparable efficacy following subcutaneous injection (Mu et al., 2018). The combination of BTZ and tannic acid, which inhibits heat shock protein 90, has also been reported to reduce the incidence of BIPN (Mitsiades et al., 2006). Furthermore, antifungal azoles have been associated with early-onset severe BIPN (Hashimoto et al., 2012).

The severity of BIPN in MM patients was strongly associated with the baseline presence of peripheral neuropathy (Dong et al., 2022). Approximately 20% of patients exhibit sensory polyneuropathy before starting BTZ treatment, which is thought to be attributed to a proteasome inhibitor-like effect (Richardson et al., 2009a). Moreover, Baseline neuropathy and comorbidities related to diabetes mellitus may also serve as predictors of the occurrence and severity of BIPN (Argyriou et al., 2014); however, the relationship between overweight/obesity, diabetes, and BIPN remains undefined (Velasco et al., 2019). According to reports, neuropathic pain is more prevalent in MM patients who have undergone prior treatment (Corso et al., 2010). Interestingly, MM patients treated with BTZ appear to experience a higher incidence of BIPN than those with solid tumors, suggesting the presence of a myeloma-specific factor that contributes to BIPN development (Broyl et al., 2010). The occurrence of BIPN may also be associated with age, race, and gender (Corso et al., 2010; Kanbayashi et al., 2010; Mateos et al., 2006; Sun L. F. et al., 2023); but still, the relationship between age and the development of BIPN remains unconfirmed in large-scale clinical trials (Richardson et al., 2009b).

Multiple genome-wide association studies (GWAS) have identified key susceptibility genes and elucidated their mechanisms of action. For example, single nucleotide polymorphisms (SNPs) in the PKNOX1 gene are not only associated with an increased risk of painful peripheral neuropathy (Magrangeas et al., 2016; Zhou X. et al., 2023), but may also influence BIPN by regulating the transcription of the pain biomarker monocyte chemoattractant protein-1 (Zhang and De Koninck, 2006). Mutations in the CBS gene alter its expression, and the CBS–hydrogen sulfide (H_2_S) signaling pathway is implicated in both neurodegenerative and inflammatory diseases (Miao et al., 2014; Wang et al., 2012), indicating its potential involvement in BIPN. Intron variants in the ASIC2 and SMOC2 genes, as well as other site-specific variants, have also been associated with BIPN (Min et al., 2024). ASIC2 is linked to mechanical pain and neuronal damage (Cheng et al., 2018; Jiang et al., 2017), whereas SMOC2 is associated with neurodegeneration and nociceptive signal transmission (Guo et al., 2024; Wojtas et al., 2024; Zhang S. et al., 2022). Other functionally relevant SNPs have been shown to influence key biological processes, including neuronal development, axonal growth, and signal transduction (Campo et al., 2018).

**FIGURE 1:**
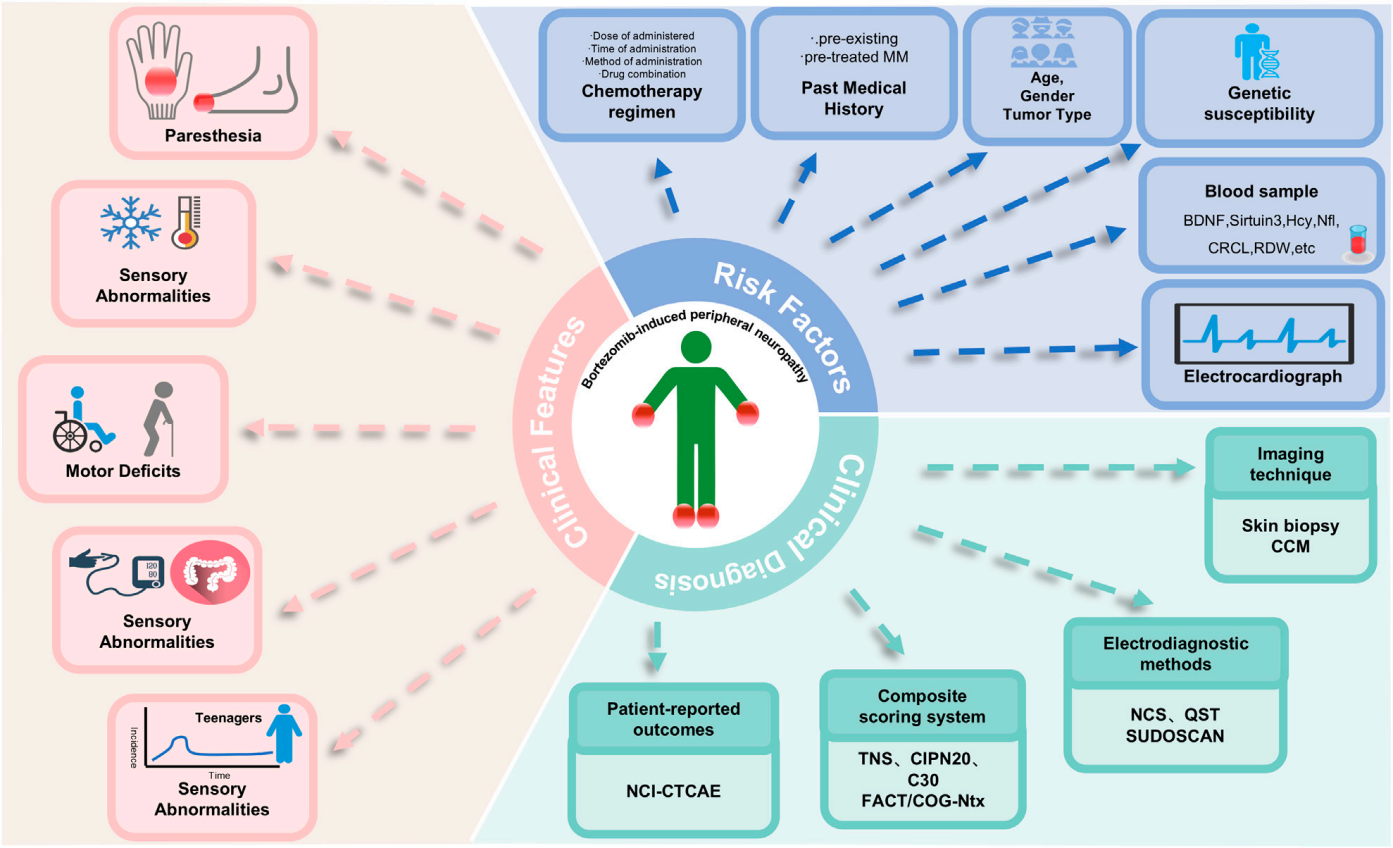
Clinical features, risk factors and clinical diagnosis of BIPN. Abbreviations: BDNF, brain-derived neurotrophic factor; CCM, Noninvasive Corneal Confocal Microscopy; CIPN20, European Organization for Research and Treatment of Cancer Quality of Life Questionnaire Chemotherapy-Induced Peripheral Neuropathy 20; CRCL, creatinine clearance; C30, European Organization for Research and Treatment of Cancer Quality of Life Questionnaire; FACT/COG-Ntx, Functional Assessment of Cancer Therapy/Gynecologic Oncology Group-Neurotoxicity; Hcy, homocysteine; NCI-CTCAE, National Cancer Institute-Common Terminology Criteria for Adverse Events; NCS, Nerve Conduction Studies; Nfl, neurofilament light chain; QST, Quantitative Sensory Testing; TNS, Total Neuropathy Score.

Other factors, including impaired renal function (indicated by abnormal creatinine clearance), are also potential risk factors (Jagannath et al., 2005; Morabito et al., 2011; San-Miguel et al., 2008). The study has demonstrated a threshold effect between red blood cell distribution width and the risk of BIPN (Ren et al., 2023). Furthermore, vitamin D deficiency has been associated with both the occurrence and severity of BIPN (Wang et al., 2016). Elevated serum sirtuin 3 levels (Yang et al., 2021),homocysteine levels (Zhang et al., 2024) and neurofilament light chain levels (Cebulla et al., 2023) are believed to be associated with BIPN. Phytohemagglutinin-induced preconditioning of whole blood IL-2 mRNA levels could potentially serve as a biomarker for predicting the onset of BIPN (Watanabe et al., 2013). Patients with BIPN exhibit decreased levels of nerve growth factor (Youk et al., 2017). Additionally, the reduced plasma levels of brain-derived neurotrophic factor (BDNF) observed in BIPN patients may be associated with BTZ-mediated inhibition of platelet aggregation and activation, which in turn leads to decreased BDNF release and consequently impairs its neuroregenerative effects on peripheral nerves (Azoulay et al., 2019). A retrospective study has found that lower R-R interval variation could also predict the occurrence of BIPN (Nishiwaki et al., 2020). Future research should further investigate the relationship between these factors and the development of BIPN (Figure 1).

## 3 Pathogenesis of BIPN

**FIGURE 2:**
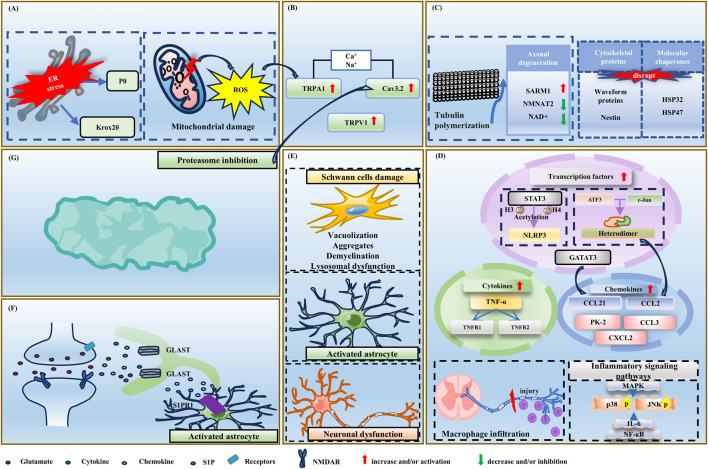
The pathogenesis of BIPN. Starting from the top-left panel and moving clockwise, the panels are: **(A)** Subcellular dysfunction and oxidative stress. **(B)** Ion channel abnormalities. **(C)** Cytoskeletal damage and axonal degeneration. **(D)** Neuroinflammation. **(E)** Schwann cells damage, astrocyte activation, and neuronal dysfunction. **(F)** Central nervous system dysfunction. **(G)** Proteasome inhibition. Abbreviations: ATF3, activating transcription factor 3; Cav3.2, voltage-gated calcium channel 3.2; CCL2, C-C motif chemokine ligand 2; CCL3, C-C motif chemokine ligand 3; CCL21, C-C motif chemokine ligand 21; CXCL2, C-X-C motif chemokine ligand 2; GATA3, GATA-binding protein 3; GLAST, glutamate-aspartate transporter; HIF1α, hypoxia-inducible factor-1α; H3, histone H3; H4, histone H4; IENF, intraepidermal nerve fibers; IL-1β, interleukin-1β; IL-6, interleukin-6; JNK, c-Jun N-terminal kinase; Krox20, early growth response-2; NAD+, nicotinamide adenine dinucleotide; NF-kB, nuclear factor-kappa B; NLRP3, NOD-like receptor protein 3; NMDAR, N-methyl-D-aspartate receptor; NMNAT2, nicotinamide mononucleotide adenylyltransferase 2; PK2, recombinant prokinetic peptide 2; P0, Peripheral Myelin P0 Protein; P2XRs, purinergic 2X receptors; ROS, reactive oxygen species; S1P, sphingosine-1-phosphate; S1PR1, recombinant sphingosine-1-phosphate receptor 1; SARM1, sterility 1 containing α and Toll/interleukin-1 receptor motifs; STAT3, signal transducer and activator of transcription 3; TNF-α, tumor necrosis factor-alpha; TRPA1, transient receptor potential ankyrin 1.

Despite substantial advancements in elucidating the potential pathogenesis of BIPN, the exact mechanisms driving its development remain inadequately understood. Therefore, we summarized the key pathways of BTZ in DRG, the dorsal horn of the spinal cord, and peripheral nerve fibers leading to the development of PN. These pathways primarily involve organelle damage, ion channel abnormalities, cytoskeletal structure and axon degeneration, neuroinflammation, cellular dysfunction, and central nervous system (CNS) dysfunction. This analysis aims to further elucidate the mechanisms underlying BIPN development (Figure 2).

### 3.1 Subcellular dysfunction and oxidative stress

#### 3.1.1 Mitochondria and oxidative stress

Mitochondrial dysfunction plays a critical role in the pathogenesis of BIPN. BTZ induces mitochondrial morphological abnormalities in the peripheral nervous system (PNS) (Cavaletti et al., 2007; Zheng et al., 2012). BTZ-treated animals have been shown to exhibit mitochondrial dysfunction, including respiratory chain damage, reduced adenosine triphosphate (ATP) production, and decreased manganese superoxide dismutase (MnSOD) activity, which leads to ATP depletion and impairs energy-dependent axonal transport. Dysfunctional mitochondria generate excessive reactive oxygen species (ROS), perpetuating a vicious cycle that further aggravates neurotoxicity (Yan et al., 2021; Zheng et al., 2012; Bilinska et al., 2013; Janes et al., 2013; Staff et al., 2013) In addition, aerobic glycolysis and high protein toxicity are associated with BIPN neurotoxicity (Jannuzzi et al., 2020; Ludman and Melemedjian, 2019a). The systemic application of ROS scavengers, including phenyl-N-tert-butyl nitro and MnSOD, attenuates BTZ-induced mechanical hypersensitivity (Duggett and Flatters, 2017).

#### 3.1.2 Endoplasmic reticulum (ER) stress

Defects in ER-associated degradation, unfolded protein responses, and ER stress have been implicated in various myelin disorders (Volpi et al., 2016). Schwann cells (SCs) in the BIPN mouse model have been shown to exhibit acute and transient ER damage. BTZ-induced ER stress suppresses the expression of key myelination-associated genes, including P0 and Krox20, in SCs, thereby disrupting their physiological functions and contributing to the demyelination of peripheral nerve fibers (Shin et al., 2010).

### 3.2 Ion channel abnormalities

Ion channel receptors play a key role in converting nociceptive stimuli into electrical signals, which are then transmitted as pain signals from peripheral nerves to the brain. Impaired ion channel activity or its imbalance is recognized as a critical factor in the onset and progression of neuropathic pain (Pozzi et al., 2024; Stevens and Stephens, 2018).

#### 3.2.1 Calcium channel

Voltage-gated calcium channel subtype 3.2 (Cav3.2) T-type calcium channels are a key target in the pathophysiology of neuropathic pain in peripheral sensory neurons (Cai et al., 2021). BTZ treatment increases the expression of ubiquitin-specific protease 5, inhibits the proteasomal degradation of Cav3.2 T-type calcium channels in mice, and elevates Cav3.2 levels in nociceptive receptors, thereby facilitating the onset of neuropathic pain (Tomita et al., 2019). The mechanical hypersensitivity caused by BTZ can be substantially reduced through the use of Ca^2+^ channel blockers, such as tramadol, pregabalin, and ethosuximide (Duggett and Flatters, 2017; Yamamoto et al., 2015).

#### 3.2.2 Transient receptor potential Channels (TRP)

TRP channels are the largest receptor family involved in detecting noxious stimuli and play a pivotal role as targets in the development of new analgesics. Several TRP channels have been recognized as potential therapeutic targets for treating CIPN (Chen et al., 2024). As a member of the TRP channel family, transient receptor potential ankyrin 1 (TRPA1) is strongly linked to the progression of BIPN (Talavera et al., 2020). Blocking interleukin-6 (IL-6) or tumor necrosis factor-alpha (TNF-α) signaling, which decreases TRPA1 protein expression, has been demonstrated to alleviate neuropathic pain in rats with BIPN (Li et al., 2018; Liu et al., 2019). TRPA1-knockout mice were protected from developing mechanical and cold pain sensitization following BTZ administration (Trevisan et al., 2013). TRPA1 is activated by a range of stimuli, such as noxious cold, chemical irritants, hypoxia, acidic conditions, and ROS (Takayama et al., 2019; Wu et al., 2010). Treatment with the TRPA1 antagonist or the antioxidant alpha-lipoic acid halted the progression of BIPN. Transient receptor potential vanilloid 1 (TRPV1) channel contribute to peripheral nerve pain by exhibiting both sensitizing and desensitizing effects on pain pathways, thus influencing the overall pain response (Gao et al., 2024). BTZ has been shown to strongly sensitize TRPV1, potentially leading to its transient activation and thereby promoting the development and maintenance of BIPN (Sprague et al., 2023).

### 3.3 Cytoskeletal damage and axonal degeneration

Cytoskeletal damage and axonal degeneration are key pathological aspects of BIPN. BTZ disrupted cytoskeletal proteins (waveform proteins and nestin) and molecular chaperones (HSP32 and HSP47), impairing neuronal cell integrity (Karademir et al., 2018). Although BTZ is a proteasome inhibitor, it significantly interferes with the dynamic balance of microtubule proteins, leading to increased microtubule protein polymerization and acetylation accumulation in *ex vivo* models (Staff et al., 2013; Cartelli et al., 2022; Meregalli et al., 2014; Pero et al., 2021; Poruchynsky et al., 2008). This imbalance in microtubule dynamics impedes dorsal root ganglion (DRG) neurite outgrowth and axonal mitochondrial transport and is accompanied by neurofilament accumulation and sensory neuron reduction within the DRG (Staff et al., 2013; Ale et al., 2015). Other proteasome inhibitors (lactacystin and MG-132) cause similar microtubule protein alterations (Staff et al., 2013; Poruchynsky et al., 2008). In addition, BTZ triggers the axonal degeneration program: decreasing axonal Nicotinamide mononucleotide adenylyltransferase 2 levels, activating Sterility 1 containing α and Toll/interleukin-1 receptor motifs (SARM1), and depleting nicotinamide adenine dinucleotide, a process regulated by caspase-mediated transcription; inhibition of SARM1 or caspases prevents axonal degeneration (Geisler et al., 2019; Snavely et al., 2022). Loss of axonal terminal plasticity may trigger distal nerve fiber degeneration and nociceptive hypersensitivity (Chen et al., 2024; Gornstein and Schwarz, 2017; Thomas et al., 2023). It is crucial to deeply investigate the association between cytoskeletal structure, axonal degeneration and BIPN.

### 3.4 Neuroinflammation

#### 3.4.1 Macrophage infiltration

The involvement of macrophages in neuropathy has been widely investigated (Msheik et al., 2022). Research has indicated that macrophage infiltration is associated with the severity of BIPN (Shin et al., 2010). Following BTZ treatment, transcript levels of macrophage chemokines CC chemokine ligand 3 and C-X-C motif chemokine ligand 2 were elevated in SCs, while CC chemokine ligand 2(CCL2) protein expression was also increased in DRG neurons (Shin et al., 2010; Kanda et al., 2006). Intravenous high-dose immunoglobulin has been shown to reduce neuro macrophage infiltration and alleviate BIPN symptoms (Meregalli et al., 2018). The prokineticin receptor antagonist PC1 downregulates macrophage activation markers, ameliorates structural damage in the PNS, prevents complete neuroimmune activation in the spinal cord, and mitigates BTZ-induced neurotoxicity (Moschetti et al., 2019).

#### 3.4.2 Cytokines/chemokines/transcription factors

BTZ markedly upregulates TNF-α expression in DRG neurons, activates TNFR1 and TNFR2 co-receptors, sensitizes nociceptive neurons, and mediates mechanical allodynia (Li et al., 2016). TNF-α antagonists or TNFR knockdown suppress c-Jun N-terminal kinase (JNK) phosphorylation and alleviate BTZ-induced mechanical hypersensitivity (Zhang et al., 2014). In addition, CCL2 and TNF-α signaling co-localize with DRG sensory neurons, and prokineticin-2 (PK2) levels are elevated in CD68-positive macrophages following BTZ treatment (Moschetti et al., 2019). Intrathecal administration of CCL2-neutralizing antibodies attenuates BTZ-induced mechanical allodynia (Liu et al., 2016). BTZ induces the expression of activating transcription factor 3 and c-Jun in DRG neurons, which form a transcriptional heterodimer that upregulates CCL2 and promotes mechanical allodynia, with dose- and time-dependent variation observed in some models (Yamamoto et al., 2015; Liu et al., 2016; Yin et al., 2019). GATA-binding protein 3 (GATA3) mediates epigenetic upregulation of C-C motif chemokine ligand 21 (CCL21) in spinal dorsal horn neurons, contributing to neuropathic pain; intrathecal administration of GATA3-targeting siRNA reduces pain (Zheng et al., 2019). BTZ enhances NLRP3 expression through signal transducer and activator of transcription 3 (STAT3)-dependent histone H3/H4 acetylation; inhibition of either STAT3 or NLRP3 *via* siRNA administration prevents the development of BTZ-induced mechanical allodynia (Liu et al., 2018).

#### 3.4.3 Inflammatory signaling pathways

The mitogen-activated protein kinase (MAPK) pathway plays a crucial role in the pathogenesis of BIPN. Phosphorylation of JNK and p38 MAPK is upregulated in DRG neurons following BTZ administration (Li et al., 2018; Liu et al., 2019; Zhang et al., 2014; Guo et al., 2020). Moreover, pharmacological inhibition of these kinases has been shown to offer protection against BIPN (Guo et al., 2020). IL-6 receptor (IL-6R) protein expression is upregulated in rat DRG following BIPN treatment, and inhibition of the IL-6 signaling pathway reduces JNK and p38 MAPK phosphorylation (Liu et al., 2019). Evidence indicates that nuclear factor κB (NF-κB) signaling can be activated in the peripheral nervous system following BTZ administration (Ale et al., 2014), and pharmacologic inhibition of NF-κB alleviates symptoms in mouse models of BIPN (Ale et al., 2016). In contrast, in multiple myeloma cells, BTZ suppresses NF-κB activity by preventing proteasome-mediated degradation of IκBα, thereby exerting antitumor effects. These observations suggest context- and time-dependent regulation of NF-κB across cellular compartments: within the injured peripheral nerve microenvironment, transient and acute activation may participate in stress responses and neuroprotection, whereas sustained activation drives neuroinflammation and axonal degeneration, promoting BIPN. Potential mechanisms include calpain-mediated alternative degradation of I-kappa-B-alpha under oxidative or calcium stress, microtubule depolymerization leading to upstream signaling, and amplification of inflammatory pathways triggered by cytokines and damage-associated molecular patterns (Ghelardini et al., 2014).

### 3.5 SCs damage, astrocyte activation, and neuronal dysfunction

#### 3.5.1 SCs damage

SCs are the primary glial cells in the PNS and play crucial roles in neurodegenerative diseases while promoting nerve regeneration (Bosch-Queralt et al., 2023; Taveggia and Feltri, 2022; Wang et al., 2022). BTZ administration may induce cytoplasmic vacuolization in SCs, and may trigger demyelination of peripheral nerves, resulting in progression of sensory deficits (Thawani et al., 2015; Bruna et al., 2010; Carozzi et al., 2013). Damage to SCs leads to primary demyelination, characterized by myelin breakdown, which ultimately results in axonal degeneration (Yan et al., 2021). Another study suggests that the formation of SCs aggregates is involved in the mechanism underlying BIPN (Watanabe et al., 2010). Recent studies have suggested that lysosomal dysfunction in SCs is linked to the pathophysiology of BIPN (Wu et al., 2023).

#### 3.5.2 Astrocyte activation

Astrocytes are implicated in the pathogenesis of BIPN (Robinson et al., 2014a; Stockstill et al., 2018). BTZ treatment activates astrocytes in the dorsal horn of the spinal cord, leading to a reduction in the expression of crucial glutamate transporter proteins, including glutamate transporter-1 and glutamate/aspartate transporter. This leads to impaired glutamate reuptake, resulting in elevated extrasynaptic glutamate concentrations and subsequent neuronal excitotoxicity (Robinson et al., 2014a; Liaw et al., 2005). Moreover, BTZ influences the metabolism of the sphingomyelin synthesis pathway in the spinal dorsal horn by enhancing the expression of sphingosine-1-phosphate (S1P) and its receptor, S1PR1 (S1P receptor 1). S1PR1 is mainly expressed in astrocytes, where it plays a key role in driving astrocyte-mediated neuroinflammation, thereby disrupting glutamatergic balance (Stockstill et al., 2018).

Inhibiting S1PR1 effectively prevented the elevated glial fibrillary acidic protein (GFAP) immunoreactivity, morphological changes in astrocytes, increased presynaptic glutamate release, and alterations in cytokine expression. Additionally, astrocyte-specific S1PR1 knockdown showed a protective effect against BIPN, suggesting that astrocytic S1PR1 plays a key role in BIPN pathogenesis (Stockstill et al., 2018).

#### 3.5.3 Neuronal dysfunction

Damage to DRG neurons is a key characteristic of neuropathic pain. BTZ induces structural damage to DRG neurons, as evidenced by marked reductions in the size of the soma, nucleus, and nucleolus (Yan et al., 2021; Meregalli et al., 2014). BTZ reduces extranuclear transcription and disrupts protein synthesis machinery by trapping polyadenylated RNA in poly(A) particle nucleosomes (Palanca et al., 2014). However, BTZ does not compromise the survival of DRG neurons (Meregalli et al., 2014). Epigenetics is strongly associated with neuropathic pain (Petho et al., 2023), and alterations in epigenetic mechanisms are implicated in the development of BIPN (Kulig et al., 2023; Luczkowska et al., 2022). Histone H3 acetylation and histone H3 lysine 9 acetylation were significantly reduced in neurons following BTZ treatment (Luczkowska et al., 2022). Prokineticin is a group of chemokines that promote the release of pro-inflammatory and pro-nociceptive mediators, and are also involved in the epigenetic regulation of genes related to cell differentiation. BTZ treatment upregulates PK2 and the epigenetic regulatory enzyme KDM6A in the PNS and spinal cord, inducing mechanical pain (Moschetti et al., 2019; Rullo et al., 2021). Additionally, blocking PK signaling has been demonstrated to prevent neurotoxicity caused by BTZ (Moschetti et al., 2020). Neuronal microRNAs miR-6810-5p and miR-672-5p play indirect roles in neuroprotection and the modulation of neuronal excitability (Luczkowska et al., 2022; Sun Y. et al., 2023). Purinergic P2X3 signaling has emerged as a promising therapeutic target for the treatment of BIPN (Holzer et al., 2022). Furthermore, BTZ treatment in rats caused a decrease in SIRT1 levels, which subsequently reduced STAT3-mediated histone hyperacetylation in the neutrophilic alkaline phosphatase 1 promoter region of dorsal horn neurons, ultimately contributing to the development of mechanical pain hypersensitivity (Chen et al., 2018).

### 3.6 Central nervous system dysfunction

BTZ does not cross the blood-brain barrier, suggesting that it may indirectly contribute to CNS dysfunction (Meregalli et al., 2014). The concentration of glutamate, the predominant neurotransmitter in cerebrospinal fluid, has been found to be elevated in BIPN rats (Ghelardini et al., 2014). The glutamate/aspartate transporter protein (GLAST) is a key extracellular glutamate transporter predominantly expressed by astrocytes. BTZ administration resulted in reduced expression of GLAST and GFAP in the dorsal horn of the spinal cord, accompanied by morphological alterations in astrocytes (Robinson and Dougherty, 2015). This suggests that the elevated glutamate concentration following BTZ administration may result from GLAST downregulation. Furthermore, co-administration with ceftriaxone prevented both the elevated glutamate concentration and BTZ-induced mechanical pain abnormalities by activating glutamate transporter activity (Robinson and Dougherty, 2015). Wide dynamic range (WDR) neuronal hyperactivity in the dorsal horn of the spinal cord is closely associated with CIPN (Meesawatsom et al., 2020). Disruption of spinal glutamate homeostasis impacts WDR neuronal firing in the spinal cord, inducing mechanical hypersensitivity (Robinson et al., 2014a). Studies have shown that BTZ can sensitize peripheral sensory nerve fibers, resulting in overactivity of WDR neurons and subsequent abnormal pain or nociceptive hypersensitivity (Carozzi et al., 2013). Additionally, in BTZ-treated neurons in the dorsal horn of the spinal cord, the frequency of miniature excitatory postsynaptic currents (EPSCs) increased without a change in amplitude, suggesting enhanced presynaptic glutamate release in an animal model of BIPN (Stockstill et al., 2018; Xie et al., 2017).

Increased phosphorylation of multiple intracellular kinases in the spinal cord, including c-Jun NH2-terminal kinase, extracellular signal-regulated kinase (ERK), protein kinase C (PKC), phosphatidylinositol-3 kinase, and p38-MAPK, is closely linked to the pathology of BIPN (Meregalli et al., 2014; Robinson et al., 2014a; Stockstill et al., 2018; Ghelardini et al., 2014; Robinson and Dougherty, 2015; Xie et al., 2017). Cytokines and chemokines in the spinal cord are essential for the development and progression of BIPN, playing a significant role in its underlying pathological mechanisms. The expression of TNF-α and CCL21 is upregulated in the dorsal horn neurons of the spinal cord, while IL-1β and PK-2 levels are increased in the astrocytes of the dorsal horn (Moschetti et al., 2019; Li et al., 2016; Zheng et al., 2019; Xie et al., 2017). BTZ reduced levels of anti-inflammatory cytokines IL-4 and IL-10 in the spinal cord (Moschetti et al., 2019; Stockstill et al., 2018). Inhibiting inflammatory cytokine signaling within the spinal cord has been shown to prevent the onset of neuropathic pain induced by BTZ (Moschetti et al., 2019; Li et al., 2016; Zheng et al., 2019; Zheng et al., 2019).

### 3.7 Proteasome inhibition

Proteasome inhibition remains a significant unresolved issue in the pathogenesis of BIPN. Intravenous injection of BTZ in rats was found to inhibit proteasome activity in the blood, sciatic nerve, and DRG, while the brain remained unaffected (Meregalli et al., 2014). Furthermore, proteasome inhibition by BTZ led to increased Cav3.2 expression, a protein implicated in BIPN; similar effects were observed with another PI, MG-132 (99). In addition, another study found that, despite having similar proteasome inhibitory effects, carfilzomib did not reduce neurite growth as BTZ did (Arastu-Kapur et al., 2011). The findings indicate that additional factors beyond proteasome inhibition contribute to the pathogenesis of BIPN. The serine protease cathepsin G was identified as a non-proteasomal target of BTZ. Moreover, BTZ treatment reduced cathepsin G activity in patient blood, potentially related to BTZ-induced protein oxidation (Jannuzzi et al., 2020; Karademir et al., 2018; Arastu-Kapur et al., 2011). Future studies should further investigate the non-proteasomal mechanisms underlying BIPN.

## 4 Prevention and treatment of BIPN

To date, neither the available treatments for BIPN nor preventive strategies have been supported by conclusive evidence. This review summarizes the drugs currently available for the treatment and prevention of BIPN, with the aim of providing insights for future drug development (Table 1).

**TABLE 1:** Summary of therapeutic approaches in BIPN.

Agent	Object	Strategy	Targets examples	Effects	NCT number/References
Analgesic drugs					
Pregabalin	MM patients	Therapeutic: regabalin (75 mg, p.o.) was administered for at least 6 months after PN symptoms appear.	—	Improved neurological symptoms	Maschio et al. (2019a)
Gabapentin	C57BL/6 NCr Mice	Therapeutic: A single administration of Gabapentin (30 and 100 mg/kg, i.g. or 30 and 100 μg/site, i.c.) was performed on 12 days after BTZ injection.	Activated the descending noradrenergic system	Reduced neural excitability and inhibited BTZ-induced mechanical allodynia	Kitamura et al. (2014)
Tramadol	SD rats	Therapeutic: A single administration of Gabapentin (10 mg/kg, i.g.) was performed on 12–15 days after BTZ injection.	μ-opioid receptor-dependent mechanism	Inhibited BTZ-induced mechanical allodynia	Yamamoto et al. (2015)
Duloxetine	SD rats	Therapeutic: A single administration of duloxetine (30 mg/kg, i.g.) was performed on 12–15 days after BTZ injection.	—	Inhibited BTZ-induced mechanical allodynia	Yamamoto et al. (2015)
Mexiletine	SD rats	Therapeutic: A single administration of mexiletine (100 mg/kg, i.g.) was performed on 12–15 days after BTZ injection	Inhibited Ca^2+^ and Na^+^ channels	Inhibited BTZ-induced mechanical allodynia	Yamamoto et al. (2015)
Amitriptyline	Patients	Therapeutic: 10% amitriptyline cream (ad us. ext.) was administered twice a day for 30 days after PN symptoms appear.	—	Reduced pain, anxiety, and depression in patients with BIPN	Rossignol et al. (2019)
Goshajinkigan	SD rats	Therapeutic: A single administration of GJG (1.0 g/kg, i.g.) was performed on 12–15days after the development of neuropathy.	κ-opioid receptor-dependent mechanism	Reduced BTZ-induced mechanical allodynia	Higuchi et al. (2015)
Controlled- release oxycodone	MM Patients	Therapeutic: CRO was initially administered (10 mg q12 h,p.o.) and adjusted stepwise (10–40 mg q12h, max 80 mg/day) over 14 days based on pain relief.	—	Reduced BTZ-induced mechanical allodynia and well tolerated	Cartoni et al. (2012)
CR4056	Wistar rats	Preventive: a. CR4056 (6 mg/kg, i.g.) was administered once daily starting from the first day after the injection of BTZ for 4 weeks. Therapeutic: b. A single administration of CR4056 (6 mg/kg,i.g.) was performed daily on 2 weeks after BTZ injection.	A new analgesic I2 ligand	Both a and b reduced bortezomib-induced mechanical allodynia	Meregalli et al. (2012)
Antioxidants					
Glutathione	MM patients	Preventive: Glutathione (2.4 g, i.v.) was administered once daily starting from the 2–3 days before chemotherapy, until the end of the chemotherapy cycle.	Reduced oxidative stress	Reduced the incidence and severity of CIPN	Huang et al. (2021)
Acetyl-L-carnitine	SD rats	Preventive: ALCAR (100 mg/ml/kg, i.g.) was administered on the day before the first injection of BTZ for a total of 22 days.	Reversed the mitochondrial dysfunction	Reversed the development of sensory neuropathy in BIPN	Zheng et al. (2012)
PBN	SD rats	Therapeutic: A single administration of PBN (100 mg/kg, i.p.) was performed on 24 days after BTZ injection	Scavenger of ROS	Decreased BTZ-induced mechanical hypersensitivity	Duggett and Flatters (2017)
Evodiamine	DRGs and SCs	Preventive: SCs or DRGs were co-incubated with EVO (2.5, 5, or 10 μM) and 20 nM BTZ for 24 h.	Inhibitor of the MAPK signal path, attenuated oxidative stress and ferroptosis	Improved BTZ-induced peripheral neurotoxicity	Tang et al. (2024)
Docosahexaenoic acid and α-lipoic acid	MM patients	Preventive: A tablet (containing docosahexaenoic acid 400 mg, α-lipoic acid 600 mg, p.o.) was administered twice daily for 6 months.	—	Prevented BIPN	Maschio et al. (2019b)
Phα1β	C57BL/6J mice	Therapeutic: A single administration of Phα1β (10–100 pmol/site, i.t.) was performed on 7 days after the injection of BTZ.	Inhibition of TRPA1	Attenuated BTZ-induced mechanical and cold hyperalgesia	Tonello et al. (2017)
Nerve-protecting drugs					
Mecobalamin	MM patients	Preventive: a. Mecobalamin (500 μg, i.v.) was administered once every other day starting from the 2–3 days before chemotherapy, until the end of the chemotherapy cycle. Preventive: b. Mecobalamin (2000 μg, i.v.) was administered once daily starting from the 1 h before chemotherapy for 28 days.	—	Reduced the incidence and severity of CIPN	a (Huang et al., 2021) b (Zhang et al., 2017)
Dexanabinol	Wistar rats	Preventive: Dexanabinol (10 mg/kg, i.p.) was co-administered with BTZ 3 times per week for 8 weeks.	Inhibitor of NMDA receptors and ROS	Prevented BTZ-induced mechanical allodynia and thermal hyperalgesia and partially restores intraepidermal nerve fiber density	Bloomingdale et al. (2021)
Minocycline	SD rats	Preventive: Minocycline (25.0 mg/kg, i.p.) was administered once daily starting from the first day before the injection of BTZ for 9 days.	Prevented astrocyte activation	Prevented BIPN	Robinson and Dougherty (2015)
Ceftriaxone	SD rats	Preventive: Ceftriaxone (150 μg, i.t.) was administered once daily starting from the first day before the injection of BTZ for 9 days.	Upregulator of glutamate transporter	Prevented BIPN	Robinson and Dougherty (2015)
FTY720	SD rats	Preventive: a. FTY720 (dose-dependent effects,0.003, 0.03, 0.1, or 0.3 mg/kg,i.g.) was co-administered with BTZ daily starting from day 0 for 5 days. Therapeutic: b. FTY720 (0.3 mg/kg, s.c.) was administered daily starting from day 24 or 25 on peak pain for 6 days.	S1PR1 functional antagonist	a. Prevented BTZ-induced neuropathic pain b. Reversed BTZ-induced neuropathic pain	Stockstill et al. (2018)
Torin1	a. C57BL/6J mice b. RSC96 cells	Therapeutic: a. Torin1 (20 mg/kg/d, i.p.) was administered daily starting from the 3rd and 4th week after the injection of BTZ. Preventive: b. Torin1 (20 μM) was co-administered for 24 h with BTZ.	Activator of lysosomal	a. Ameliorated BIPN b. Reversed BTZ-induced lysosome dysfunction	Wu et al. (2023)
2-amino-5-phosphonopentanoic acid	a. Spinal cord slices b. SD rats	Therapeutic: a. Pre-incubation of spinal cord slices with 50 μM AP5 for 60 min before recording. Therapeutic: b. A single administration of AP5 (1 µg or 10 μg, i.t.) was performed on 7–10 days after the final BTZ injection.	Antagonist of NMDAR	a. Reduced frequency of mEPSCs and amplitude of evoked EPSCs in BTZ-treated rats b. Decreased BTZ-induced mechanical allodynia and hyperalgesia	Xie et al. (2017)
Monosialotetr-ahexosylgangl-ioside	MM patients	Preventive: GM (100 mg, i.v.) was administered once at day1–2, 8–9, 15–16, 22–23	Nerve-protecting drug	Reduced BIPN	NCT02093910: Completed
Paeoniflorin	a. PC12 Cells b. C57BL/6J mice	Preventive: a. Paeoniflorin (20 μM) was co-administered for 24 h with BTZ. Preventive: b. Paeoniflorin (50 mg/kg, i.g.) was administered once daily starting from the first day after the first injection of BTZ for 25 days.	Reduced IL6 levels and regulated PARKIN-mediated mitochondrial autophagy	Decreased BIPN	Sun et al. (2022)
Guizhi Fuling capsules	a. MM patients b. PC12 Cells c. C57BL/6J mice	Preventive: a. Guizhi Fuling capsules three capsules, p.o.) was administered three times daily from the first day after the first injection of BTZ for 4 weeks. Preventive: b. 50 nM BTZ + 20% drug-containing serum for 24 h. Preventive: c. Guizhi Fuling capsules (420 mg/kg, i.g.) was administered once daily from the first day after the first injection of BTZ for 25 days.	Activated the mTOR pathway to detect block autophagy	Ameliorated BIPN	Fu et al. (2025)
Others drugs					
Immunoglobulins	Wistar rats	Preventive: a. Immunoglobulins (1 g/kg, i.v.) was administered every 2 weeks from the beginning of BTZ treatment Therapeutic: b. Immunoglobulins (1 g/kg, i.v.) was administered after 4 weeks of BTZ treatment (therapeutic schedule) for a total of 8 weeks.	Reduced nerve macrophage infiltration	Reduced BTZ-induced heat and mechanical allodynia	Meregalli et al. (2018)
Ethosuximide	SD rats	Preventive: Ethosuximide (200 mg/kg, i.p.) was administered starting from 21 days after first injection of BTZ.	Inhibitor of T-type calcium-channel	Decreased BTZ-induced mechanical hypersensitivity	NCT04431778: Completed/ Duggett and Flatters (2017)
HC030031	C57BL/6J mice	Therapeutic: HC030031 (300 mg/kg, i.g.) was administered starting from the day 7 after the injection of BTZ.	Inhibition of TRPA1	Alleviated BTZ-induced mechanical and cold hyperalgesia	Trevisan et al. (2013)
Tamoxifen	Balb/c mice	Preventive: Tamoxifen (30 mg/kg, i.g.) was administered starting from the 12 h after the injection of BTZ.	Inhibitor of the PKC—ERK pathway	Suppressed BTZ-induced cold and mechanical allodynia	Tsubaki et al. (2018b)
Chelerythrine	SD rats	Therapeutic: a. Pre-incubation of spinal cord slices with 10 μM chelerythrine for 60 min before recording. Therapeutic: b. A single administration of chelerythrine (1 µg or 10 µg,i.t.) was performed on 7–10 days after the final BTZ injection.	Inhibition of PKC	a. Fully reversed increased mEPSC frequency and evoked EPSC amplitude induced by BTZ b. Decreased BTZ-induced mechanical allodynia and hyperalgesia	Xie et al. (2017)
PC1	C57BL/6J mice	Therapeutic: PC1(150 μg/kg, s.c.) was administered daily from the 14 days after the injection of BTZ for 14 days.	PK receptor antagonist	Counteracted BTZ-induced-allodynia and hyperalgesia	Moschetti et al. (2019)
Trametinib	Balb/c mice	Preventive: Trametinib was administered (0.5 mg/kg, i.g.) daily starting from the 12 h after Chemotherapy for 15 days.	Inhibition of ERK 1–2 activation	Inhibited BTZ-induced cold and mechanical allodynia	Tsubaki et al. (2018a)
Lafutidine	MM patients	Preventive: Lafutidine (10 mg, p.o.) was administered twice daily after the first injection of BTZ throughout the study period.	H2-blocker	Reduced Suppressed BTZ-induced neurotoxicity	JPRN-UMIN000022073 Tsukaguchi et al. (2013)
Rapamycin	C57BL/6J mice	Therapeutic: a. A single administration of rapamycin (10 mg/kg, i.p.) was performed on 14 days after the final BTZ injection. Therapeutic: b. A single administration of rapamycin (10 nmol–10 μmol, i.t.,100 nmol transient effect; higher doses prolonged analgesia) was performed on 14 days after the final BTZ injection.	Inhibitors of mTOR	Exhibited considerable analgesia in a dose-dependent manner	Suzuki et al. (2023)
Everolimus	C57BL/6J mice	Therapeutic: a. A single administration of everolimus (30 mg/kg, i.p.) was performed on 14 days after the final BTZ injection. Therapeutic: b. A single administration of everolimus (20 nmol–20 μmol, i.t., 200 nmol transient; 2–20 μmol prolonged) was performed on 14 days after the final BTZ injection.	Inhibitors of mTOR		Suzuki et al. (2023)
Metformin	a SD rats b ICR mice c SD rats	Preventive: a Metformin (50 μg/10 μL, i.t.) was co-administered with BTZ for 10 days. Preventive: b Metformin (100 μg/kg, i.g.) was co-administered with BTZ for 5 days. Preventive and Therapeutic: c Metformin (50 mg/kg, i.p.) was co-administered with BTZ for 5 days (Preventive); Metformin (50 mg/kg, i.p.) was administered after the 6–12 days injection of BTZ for 7 days (Therapeutic use).	a Inhibited the RAGE—STAT3 pathway b Regulated AMPK—mTOR axis c Upregulated GATA3 and activated AMPKa2 and	a Attenuated BTZ-induced mechanical allodynia b Prevented BTZ-induced mechanical allodynia c Prevented and treated BTZ-induced mechanical allodynia	a (Wei et al., 2017) b (Ludman and Melemedjian, 2019b) c (Liu et al., 2022)
Non-pharmacological studies					
Acupuncture	MM patients	Therapeutic:a Acupuncture was performed three times weekly for 4 weeks, paused during week 5, then continued twice weekly for 4 more weeks (20 total treatments over 9 weeks). Therapeutic:b Acupuncture was administered twice weekly for 2 weeks, once weekly for 4 weeks, and then biweekly for 4 weeks (totaling 10 treatments over 10 weeks). c/	—	Promotes the movement of qi and blood in the diseased area	a NCT00891618: Completed/(Terpos et al., 2015) b NCT01541644: Completed/(Parasrampuria et al., 2020) c NCT04770402 Terminated
Cryocompression therapy	MM patients	Therapeutic: Daily cryocompression therapy throughout the study period.	—	Reduces neuropathy induced by BTZ hemotherapy	NCT03870451: Terminated
Repetitive transcranial magnetic stimulation	MM patients	Therapeutic: rTMS was administered once daily (14 min/session), five times per week, for 6 weeks per treatment course	—	Alleviated CIPN symptoms in MM patients	Han et al. (2017)

Abbreviations: ALCAR, Acetyl-L-carnitine; AP5, 2-amino-5phosphonopentanoic acid; BIPN, bortezomib-induced peripheral neuropathy; CIPN, chemotherapy-induced peripheral neuropathy; CRO, controlled-release oxycodone; DRGs, dorsal root ganglion neurons; EPSCs: excitatory postsynaptic currents; ERK1/2, extracellular-regulated kinase 1/2; FTY720, fingolimod hydrochloride; H2-blocker, histamine type-2, receptor antagonist; MAPK, mitogen-activated protein kinase; MM, multiple myeloma; GM, monosialotetrahexosylganglioside; GJG, goshajinkigan; mTOR, mammalian target of rapamycin; PK, receptor, prokineticin receptor; PN, peripheral neuropathy; rTMS, repetitive transcranial magnetic stimulation; SCs, RSC96 Schwann cells; SD, Sprague-Dawley; S1PR1, sphingosine-1-phosphate receptor 1; TNF α, tumor necrosis factor-alpha; TRPA1, transient receptor potential ankyrin 1.

### 4.1 Pharmacological treatment

#### 4.1.1 Analgesic drugs

Several analgesics, including duloxetine, gabapentin, pregabalin, lamotrigine, amitriptyline, and nortriptyline, have been evaluated in randomized controlled trials and open-label studies for their effectiveness in treating neuropathy (Barohn et al., 2021; Lambru et al., 2021; Lipone et al., 2020; Velasco et al., 2021; Viel et al., 2021). Some of these analgesics have demonstrated efficacy in treating BIPN. Pregabalin attenuates mechanical pain abnormalities in rats with BIPN and is frequently utilized in clinical settings (Yamamoto et al., 2015; Maschio et al., 2019a). Gabapentin, tramadol, duloxetine, and mexiletine have demonstrated efficacy in reversing BTZ-induced mechanical pain abnormalities (Yamamoto et al., 2015; Kitamura et al., 2014); in contrast, preclinical models of BIPN did not demonstrate anti-allodynic effects with diclofenac and amitriptyline treatment (Yamamoto et al., 2015). Nevertheless, clinical studies have shown that topical 10% amitriptyline cream can alleviate BTZ- or oxaliplatin-induced neuropathic pain, with a favorable safety profile (Rossignol et al., 2019). Goshajinkigan is a traditional Chinese herbal medicine used to treat limb pain, BIPN and other conditions by alleviating BTZ-induced mechanical pain abnormalities through a κ-opioid receptor-dependent mechanism (Higuchi et al., 2015). Additionally, oral controlled-release oxycodone alleviated BIPN-induced mechanical pain, was well tolerated (Cartoni et al., 2012). Furthermore, preclinical models showed that CR4056 as a novel I2 ligand analgesic was highly effective against BTZ-induced neuropathy in rats (Meregalli et al., 2012). The ASCO guidelines identify duloxetine as the sole recommended treatment for CIPN, particularly in cases associated with oxaliplatin and paclitaxel, due to its proven efficacy in alleviating neuropathic symptoms (Geraldes et al., 2024; Hershman et al., 2014; Loprinzi et al., 2020; Meng et al., 2019).

#### 4.1.2 Antioxidants

Antioxidant treatments have been shown to alleviate BTZ-induced neuropathy (Iijima et al., 2020; Zhou L. et al., 2023). Acetyl L-carnitine reverses mitochondrial dysfunction and the progression of sensory neuropathy in BIPN model rats (Zheng et al., 2012). The active oxygen scavenger phenyl‐N‐tert‐butylnitrone prevents and reverses BTZ-induced pain abnormalities (Duggett and Flatters, 2017). Evodiamine alleviates BIPN by reducing oxidative stress and ferroptosis through the inhibition of the MAPK signaling pathway (Tang et al., 2024). Clinical studies suggest that the administration of nutritional supplements, such as docosahexaenoic acid and alpha-lipoic acid, either during the 6 months prior to or concurrently with BTZ treatment, can reduce the onset and progression of BIPN. Importantly, this approach does not negatively impact patient functional autonomy or quality of life, thereby minimizing the need for interruptions in BTZ therapy (Maschio et al., 2018; Maschio et al., 2019b). Nevertheless, α-lipoic acid, which possesses antioxidant properties, could potentially disrupt the antitumor efficacy of BTZ in melanoma cells (Takacs et al., 2020). Therefore, recommending strategies to mitigate BIPN without interfering with the antitumor mechanism of BTZ remains challenging. Dimethyl fumarate and its metabolite monomethyl fumarate have been shown to attenuate BTZ-induced neuronal synapse injury through a mechanism potentially linked to the Nrf2-mediated antioxidant stress response (Kawashiri et al., 2018). Glutathione also has some therapeutic potential (Huang et al., 2021). Additionally, oxidative stress-induced activation of TRPA1 represents a promising therapeutic target. TRPA1 is implicated in CIPN, and TRPA1 blockade effectively prevents BIPN and other forms of CIPN (Trevisan et al., 2013; Nassini et al., 2015; Tonello et al., 2017). Moreover, TRPA1 has emerged as a potential target for treating both pathological pain and respiratory disorders (Moran, 2018; Mukhopadhyay et al., 2016). Future clinical trials may explore novel TRPA1-targeted analgesics.

#### 4.1.3 Targeting nerve damage and glial dysfunction

A single-center, randomized clinical trial suggests that methylcobalamin was administered may reduce the incidence of BIPN (Zhang et al., 2017). Astrocyte activation is closely associated with the development of BIPN, as previously mentioned. The neuroglial inhibitor minocycline prevents BIPN by blocking BTZ-induced astrocyte activation (Robinson et al., 2014b; Tsubota et al., 2021). Ceftriaxone has been shown to reduce the symptoms of BIPN, indicating that regulating glutamate homeostasis could represent a promising therapeutic strategy (Robinson and Dougherty, 2015). The S1P-S1PR1 signaling pathway in astrocytes plays a role in BIPN, and the FDA-approved drug Fingolimod has demonstrated efficacy in both preventing and reversing BTZ-induced neuropathic pain (Stockstill et al., 2018). However, since S1PR1 antagonism has only proven effective in male rodent models of BIPN, it is crucial to account for potential sex differences in the protective effects of Fingolimod (Stockstill et al., 2020). The lysosomal agonist Torin1 reportedly rescues demyelination and nerve conduction, reducing mechanical nociceptive sensitization in BIPN mice without affecting BTZ’s inhibition of MM cells *in vitro* (Wu et al., 2023). Paeoniflorin also inhibits neuroinflammation and improves BIPN by decreasing IL-6 levels and modulating PARKIN-mediated mitochondrial autophagy (Sun et al., 2022). A recent study has shown that Guizhi Fuling capsules can alleviate BIPN by alleviating high levels of IL-6 to regulate mTOR pathway-induced autophagy (Fu et al., 2025). Spinal cord levels of the N-methyl-D-aspartate receptor (NMDAR) is closely linked to synaptic plasticity involved in neuropathic pain development. Upon the administration of BTZ, a notable increase in the baseline frequency of miniature EPSCs was observed. This effect was later reduced by the administration of the NMDA receptor antagonist, 2-amino-5-phosphonovaleric acid (Xie et al., 2017). Systemic pharmacological treatment with dexamethasone (an NMDAR and ROS inhibitor) was found to prevent neuronal apoptosis in BIPN (Bloomingdale et al., 2021).

#### 4.1.4 Additional pharmacological studies

Studies have demonstrated that ethosuximide as a T-type calcium channel inhibitor effectively alleviates abnormal pain in BIPN (Duggett and Flatters, 2017); consistent with this finding, the expression of T-type calcium channels in DRG neurons was upregulated after BTZ administration (Tomita et al., 2019). Furthermore, two ongoing clinical trials are investigating the effects of ethosuximide on neuropathy (NCT04431778) and visceral pain (NCT02973542). Additionally, the selective T-type calcium channel modulator suvecaltamide has shown efficacy in reversing BIPN in preclinical models (Meregalli et al., 2021).

Tamoxifen and trametinib have been shown to inhibit BIPN by antagonizing the PKC and ERK, respectively (Tsubaki et al., 2018a; Tsubaki et al., 2018b). Considering that chemotherapy regimens typically involve multiple agents, the combination of standard treatments with PKC or ERK inhibitors could potentially enhance BIPN outcomes in clinical practice. Preclinical models have demonstrated that PKC inhibition by leucovorin completely reversed the elevated frequency and amplitude of miniature EPSCs in BIPN model rats. Additionally, intrathecal administration of leucovorin significantly reduced BTZ-induced mechanical pain abnormalities and nociceptive sensitization (Xie et al., 2017). Moreover, inhibiting the mechanistic target of rapamycin has been demonstrated to decrease the incidence of BIPN in preclinical models (Suzuki et al., 2023). Lafutidine as a H2-blocker has protective effects of for BIPN (Tsukaguchi et al., 2013).

Metformin has been proposed as a potential treatment for BIPN. Research indicates that BIPN may arise from the activation of the RAGE/STAT3 signaling pathway in the dorsal horn, which is driven by the accumulation of methylglyoxal. Intrathecal metformin injection significantly reduced methylglyoxal levels and RAGE upregulation in the dorsal horn of the spinal cord in a rat model of BIPN, effectively blocking BTZ-induced central sensitization and mechanical pain abnormalities (Wei et al., 2017). Additionally, it has been shown that metformin can prevent the development of neuropathic pain in BTZ models by suppressing the expression of HIF1α (Ludman and Melemedjian, 2019b). Furthermore, metformin may reduce BTZ-induced behavioral hypersensitivity by regulating AMPK2-mediated autophagy in the dorsal horn of the spinal cord in BIPN rats (Liu et al., 2022).

### 4.2 Non-pharmacological treatments

Given the limited evidence for effective pharmacologic treatments of BIPN, adjusting dosage, timing, and administration route remains the primary strategy for reducing its incidence (Richardson et al., 2012). The incidence of BIPN can be decreased by adjusting the dosage (1.3–1.0–0.7 mg/m^2^), reducing the treatment frequency (from twice weekly to once weekly), and altering the administration route (from intravenous to subcutaneous) (Dimopoulos et al., 2011; Cook et al., 2021; Hoff et al., 2024; Mateos et al., 2020; Moreau et al., 2017; Parasrampuria et al., 2020; Terpos et al., 2015). A twice-weekly BTZ dosing schedule remains the preferred option for patients with renal insufficiency or extensive bone disease (Terpos et al., 2015). Several studies indicate that acupuncture alleviates various BIPN symptoms, particularly numbness, tingling, cold sensitivity, and discomfort in the hands and feet; additionally, objective measures remain lacking (Bao et al., 2014; Garcia et al., 2014; Lyu et al., 2024; Zhi et al., 2018). A randomized controlled study demonstrated that acupuncture combined with methylcobalamin is more effective in treating BIPN than methylcobalamin alone (Han et al., 2017). A clinical registry trial (NCT03870451) indicated that cryocompression therapy could be an effective treatment for MM patients with BIPN who have undergone a BTZ-based regimen in the past. Additionally, obesity elevates the risk of developing BIPN, highlighting the importance of managing body mass index (Moore et al., 2020). Early studies indicate that repetitive transcranial magnetic stimulation is safe and effective in reducing CIPN in MM patients (Yan et al., 2023).

## 5 Conclusion and prospect

Although peripheral neuropathy is a common side effect, BTZ remains a first-line treatment for MM. In addition, the second-generation PI carfilzomib and the only oral drug ixazomib have been approved, and if they can demonstrate the same antitumor efficacy as BTZ, then PN may become a minor issue in the treatment of MM, resulting in an improved quality of life for the patient and a reduced financial burden (Beijers et al., 2016; Bonnet and Moreau, 2017; Facon et al., 2021; Perrot et al., 2024; Rifkin et al., 2024; Song et al., 2019; Strifler and Knop, 2018; Kumar et al., 2025). However, carfilzomib is linked to significant cardiac and renal toxicity, requiring a thorough evaluation of the risks and benefits of various treatment regimens in clinical practice (Yu et al., 2024). The major methods for treating BIPN at the moment are dosage modification, fewer doses, and altered routes of administration. In the interim, effective management of BIPN hinges on ongoing surveillance, early detection, and prompt intervention. Hence, it is advised to strictly follow clinical practice guidelines and conduct a risk assessment for PN before administering potentially neurotoxic medications to MM patients (Richardson et al., 2012). Although certain indicators have been associated with the development of BIPN in clinical studies, genetic variations in drug response must also be taken into account. In addition, research on myeloma-specific influences on BIPN remains limited, and the causal pathway has not been delineated. Therefore, multiple large-scale prospective studies are urgently needed to define the specific relationships involved. In parallel, combining MM mouse models with BIPN models will allow controlled evaluation of myeloma-related microenvironmental cues and disease-derived mediators on neuronal injury, thereby clarifying whether and how myeloma-specific biology modulates the onset and progression of BIPN. Identifying markers that accurately and objectively reflect the severity of BIPN is also essential. A recent small-sample trial identified specific lipid species as potential biomarkers for BIPN through lipidomic analysis of patient sera. Several lipids, including phosphatidylcholine, ceramides, neutral lipids, and oxidized fatty acids, were found to correlate with BIPN severity; but still, additional studies are required to confirm these associations (Maekawa et al., 2019).

Limited clinical trials targeting the mitigation of BIPN are grounded in preclinical data; thus, applying these findings in clinical practice could lead to more effective treatment options for patients (Bouchenaki et al., 2021). Moreover, peripheral neuropathy induced by different chemotherapeutic agents appears to share several common pathophysiological mechanisms. The S1P-S1PR1 signaling pathway is implicated in peripheral neuropathy induced by oxaliplatin, paclitaxel, and BTZ (Stockstill et al., 2018; Stockstill et al., 2020; Janes et al., 2014). Two ongoing clinical trials (NCT03941743 and NCT03943498) are investigating whether fingolimod, an S1PR1 receptor modulator, can mitigate paclitaxel-induced PN. Studies suggest that PN induced by vincristine, paclitaxel, and BTZ is linked to NAD depletion, and increasing NAD levels may prevent or reverse CIPN (Geisler et al., 2019; Hamity et al., 2017; Lococo et al., 2017). The efficacy of nicotinamide riboside, a precursor of NAD, in preventing CIPN is under investigation (NCT04112641). Thus, investigating the efficacy of fingolimod and nicotinamide riboside for BIPN represents a promising direction for future clinical research. Transporter proteins implicated in neuropathy development induced by oxaliplatin and paclitaxel have been identified (Huang et al., 2020; Leblanc et al., 2018; Sprowl et al., 2016); even so, the specific transporter proteins contributing to BTZ accumulation in the DRG remain unclear (Stage et al., 2021). Identifying the specific transporter proteins responsible for BTZ accumulation and their cell-specific effects constitutes a promising strategy. Additionally, BTZ treatment has been shown to induce macrophages to release cysteine-dependent high mobility group protein 1, which activates or accelerates RAGE and CXCR4 signaling, thereby contributing to BIPN development (Tsubota et al., 2021; Araldi et al., 2024). Thus, neuro-immune interactions, particularly between injured sensory neurons and immune cells, are emerging as a promising area of research (Ollodart et al., 2024). Peripheral neuropathy induced by chemotherapeutic agents such as paclitaxel, oxaliplatin, cisplatin, and vincristine exhibits epigenetic remodeling within sensory pathways, characterized by disrupted histone acetylation and elevated histone lactylation. These alterations drive transcriptional reprogramming, impair mitochondrial quality control, and amplify neuroimmune signaling. Interventions using histone deacetylase inhibitors or strategies that suppress histone lactylation mitigate neuropathy in preclinical models, indicating that histone-modifying pathways may serve as potential biomarkers and therapeutic targets (Zhang J. et al., 2022; Luo et al., 2025; Wang et al., 2020; Ho et al., 2025; Ma et al., 2019). Therefore, further investigation into the relationship between histone-related genes and BIPN is warranted.

As previously discussed, several pharmacological and non-pharmacological interventions, including analgesics, antioxidants, and neuroprotective agents, have shown potential benefits in managing BIPN. However, robust clinical evidence remains limited, and further studies are required to confirm their efficacy and safety. Although preclinical research has produced encouraging results, translating these findings into clinical practice remains highly challenging. Most promising therapeutic approaches for BIPN have demonstrated efficacy primarily in rodent models, while their benefits in humans are still uncertain. This translational gap can be attributed to interspecies differences, the complexity and heterogeneity of disease models, and the limited ability of current models to fully reproduce the multifactorial nature of BIPN in patients with multiple myeloma. Future studies should focus on refining experimental models, integrating multi-omics approaches, and establishing standardized assessment systems to enhance the clinical relevance of preclinical findings and facilitate the translation of novel therapeutic strategies into practice. Ultimately, individualized treatment plans developed in accordance with clinical guidelines and combining pharmacological with non-pharmacological approaches may provide more effective and patient-centered management of BIPN.

In conclusion, this review provides a summary of the incidence, risk factors, molecular mechanisms, and potential treatments for BIPN. Despite active research efforts, no targeted or curative strategies have been developed, and the prevalence of BIPN is still high. Therefore, further research into the pathogenesis and detailed characterization of BIPN could facilitate the development of innovative therapeutic agents.
